# HnRNP L and hnRNP LL antagonistically modulate PTB-mediated splicing suppression of *CHRNA1* pre-mRNA

**DOI:** 10.1038/srep02931

**Published:** 2013-10-14

**Authors:** Mohammad Alinoor Rahman, Akio Masuda, Kenji Ohe, Mikako Ito, David O. Hutchinson, Akila Mayeda, Andrew G. Engel, Kinji Ohno

**Affiliations:** 1 Division of Neurogenetics, Center for Neurological Diseases and Cancer, Nagoya University Graduate School of Medicine, Nagoya, Aichi, Japan; 2 Department of Neurology, Mayo Clinic, Rochester, Minnesota, U. S. A.; 3 Department of Neurology, Auckland City Hospital, Auckland, New Zealand; 4 Division of Gene Expression Mechanism, Institute for Comprehensive Medical Science (ICMS), Fujita Health University, Toyoake, Aichi, Japan

## Abstract

*CHRNA1* gene, encoding the muscle nicotinic acetylcholine receptor alpha subunit, harbors an inframe exon P3A. Inclusion of exon P3A disables assembly of the acetylcholine receptor subunits. A single nucleotide mutation in exon P3A identified in congenital myasthenic syndrome causes exclusive inclusion of exon P3A. The mutation gains a *de novo* binding affinity for a splicing enhancing RNA-binding protein, hnRNP LL, and displaces binding of a splicing suppressing RNA-binding protein, hnRNP L. The hnRNP L binds to another splicing repressor PTB through the proline-rich region and promotes PTB binding to the polypyrimidine tract upstream of exon P3A, whereas hnRNP LL lacking the proline-rich region cannot bind to PTB. Interaction of hnRNP L with PTB inhibits association of U2AF^65^ and U1 snRNP with the upstream and downstream of P3A, respectively, which causes a defect in exon P3A definition. HnRNP L and hnRNP LL thus antagonistically modulate PTB-mediated splicing suppression of exon P3A.

In higher eukaryotes, alternative splicing enables precise regulations of gene expression with a limited number of genes. Recent reports reveal that ∼95% of human genes undergo alternative splicing^1^. Differential pre-mRNA splicing is cooperatively coordinated by *cis*-elements comprised of exonic/intronic splicing enhancers/silencers (ESEs, ISEs, ESSs, and ISSs) and *trans*-factors that are tightly regulated in a tissue-specific and developmental stage-specific manner. The biogenesis of ribonucleoprotein complexes (RNPs) is thus coordinated with high fidelity^2^ to ensure that correct complements of RNA and proteins are present in the right cell at the right time. Mutations that impair formation of functional spliceosomes by disrupting the *cis*-elements, or by compromising RNA-binding or catalytic function of *trans*-factors can be deleterious to cells often cause human disease^2^,^3^.

Congenital myasthenic syndromes (CMSs) arise from defects in genes coding for presynaptic, synaptic, and postsynaptic proteins at the neuromuscular junction (NMJ)^4^,^5^. Most CMSs are postsynaptic and most of these are caused by recessive mutations in the acetylcholine receptor (AChR) subunit genes. *CHRNA1*, encoding the AChR α subunit, harbors an alternatively spliced 75-nt inframe exon P3A between exons 3 and 4 (Fig. 1b)^6^. Only the transcript without exon P3A, P3A(−), encodes a functional α subunit that incorporates into functional AChR at the endplate^7^. The transcript with exon P3A, P3A(+), harbors 25 extra amino acids and is inserted between codons 58 and 59 in the extracellular domain of the α subunit. Formation of a pentameric AChR starts with dimerization of the αδ subunits and of the αε subunits via the extracellular domain of each subunit^8^. Disruption of the extracellular domain by exon P3A is predicted to prevent formation of the αδ and αε dimers^9^. Exon P3A is alternatively spliced in humans, gorillas, chimpanzees, and orangutans, but not in rhesus monkeys, gibbons, mandrills, marmosets, dogs, and cats^10^,^11^. In human skeletal muscle, the P3A(−) and P3A(+) transcripts are generated in a 1∶1 ratio^12^. The P3A(+) transcript is also expressed in the normal and in nonneoplastic thymus glands of myasthenic patients, but is absent^13^ or rarely expressed^14^ in thymomas. The functional significance of the P3A(+) transcript in muscle or in the thymus gland has not been elucidated to date. We previously reported that splicing regulators, hnRNP H^15^ and polypyrimidine tract-binding protein (PTB)^16^ bind to intron 3 upstream of exon P3A and suppress inclusion of exon P3A.

HnRNP L is an abundant nuclear protein that has been identified as a global splicing regulator^17^. In addition to its important function in alternative splicing^18^,^19^,^20^,^21^, hnRNP L also plays pivotal roles in polyadenylation, in export of mRNA from genes lacking introns^22^, in internal ribosome entry site (IRES)-mediated translation^23^, and in mRNA stability^24^. Recently, hnRNP L-like, also known as hnRNP LL, a closely related paralogue of hnRNP L, has also been identified as a regulator of alternative splicing in activated T cells^25^.

In a severely affected CMS patient, we have identified a critical mutation in exon P3A that causes exclusive inclusion of exon P3A in patient muscle. Here we demonstrate a fine modulating mechanism to promote either skipping or inclusion of exon P3A, which is mediated by similar, but antagonistic, hnRNP L and hnRNP LL factors. Remarkably, presence or absence of the proline-rich region (PRR) in hnRNP L and hnRNP LL, respectively, is a crucial determinant to trigger the following splicing repression system mediated by PTB.

## Results

### Missense and pseudo-missense mutations are detected in CMS

A 53-year-old man had severe myasthenic symptoms involving all voluntary muscles since birth, a decremental electromyographic response, and no circulating anti-AChR antibodies. He responded partially to combined treatment with anticholinesterase medications and 3,4-diaminopyridine. His parents were not consanguineous and he had no similarly affected relatives.

**Figure 1:**
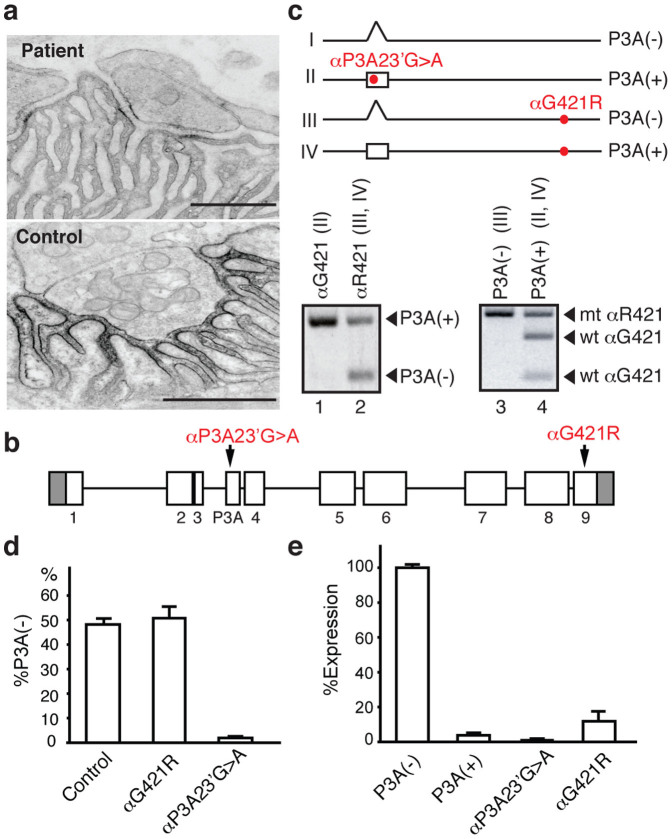
Ultrastructure of the patient endplate, identified mutations, and their functional consequences. (a) AChR expression at the patient and control endplate visualized with peroxidase-labeled α-bungarotoxin. Note restricted distribution and attenuated expression of AChR at the patient endplate. Bars = 1 μm. (b) Structure of *CHRNA1* gene and two identified mutations. (c) Four possible transcripts in muscle of the patient. Only transcript I can make a normal α subunit. A segment spanning exon P3A of transcripts from αG421- and αR421-alleles is specifically amplified by allele-specific RT-PCR using patient muscle. Transcript I is not detectable (lanes 1 and 2). P3A(−) and P3A(+) transcripts are specifically amplified by allele-specific RT-PCR of patient muscle. Nested RT-PCR products spanning αG421R are digested by NlaIV, which cuts only the wild-type αG421 fragment (lane 4) and leaves the mutant αR421 fragment undigested (lanes 3 and 4). The P3A(−) transcript almost exclusively arises from an allele with αR421 (transcript III) (lane 3), whereas P3A(+) transcripts arise from both alleles (transcripts II and IV) (lane 4). Again, transcript I is not detectable (lanes 3 and 4). (d) Allele-specific real-time RT-PCR of patient muscle. The αP3A23′G > A allele barely generates P3A(−) transcript. (e) Expression of AChR on the HEK293 cell surface introduced with the indicated α cDNAs along with the wild-type β, δ, and ε cDNAs. The expression level of αG421R-AChR is 14.7 ± 5.1% of normal (mean ± SD, n = 3).

An intercostal muscle biopsy was obtained at age 41. On fluorescent microscopy, patient endplates (EPs) showed preserved expression of acetylcholinesterase and highly attenuated expression of AChR. On electron microscopy, the structural integrity of the junctional folds and nerve terminals was preserved but some postsynaptic regions were simpler than normal. Ultrastructural localization of AChR with peroxidase-labeled α-bungarotoxin revealed marked decrease in the density and distribution of AChR on the junctional folds (Fig. 1a). The AChR index (defined as the length of the postsynaptic membrane reacting for AChR normalized for the length of the primary synaptic cleft) was reduced to ∼29% of normal (Table 1). The amplitude of the miniature EP potentials (MEPPs) was reduced to ∼23% of normal (Table 1). The number of quanta released by nerve impulse was normal. The safety margin of neuromuscular transmission in the patient is thus compromised by the AChR deficiency.

Direct sequencing of *CHRNA1*, *CHRNB1*, *CHRND*, and *CHRNE* genes encoding the AChR α, β, δ, and ε subunits, respectively, revealed two heterozygous mutations in *CHRNA1* (Fig. 1b). The G-to-A mutation at nucleotide position 1261 predicts a glycine-to-arginine substitution at codon 421 in the fourth transmembrane domain of the α subunit (αG421R). The amino acid, αG421, was shared among all the human AChR subunits and it is also perfectly conserved in the α subunit across all vertebrate species (Fig. S1a and b). αG421R is not present in 200 normal alleles or in available SNP databases (dbSNP build 137, the 1000 Genomes Project, and the NHLBI ESP). When we transfected the AChR α subunit cDNA harboring G421R mutation along with wild-type β, δ, and ε subunit cDNAs into HEK293 cells, we found that the expression level of αG421R-AChR was reduced to ∼15% (Fig. 1e), which underscored the pathogenicity of the αG421R mutation.

Because loss-of-function mutations in AChR subunit genes are generally recessive and individuals carrying a null mutation on a single allele are always asymptomatic, we looked for the second loss-of-function mutation in *CHRNA1*. We could not find any mutation except for the candidate G-to-A substitution at the 23rd nucleotide of the αP3A exon (αP3A23′G>A), which predicts an arginine-to-histidine substitution at the 8th codon in exon P3A (Fig. 1b). The αP3A23′G>A mutation was not present in 200 normal alleles or in available SNP databases. We traced αP3A23′G>A and αG421R changes in family members, and found that these two mutations are heteroallelic and recessive (Fig. S1c). We first assumed that αP3A23′G>A was a rare polymorphism, because a wild-type transcript carrying exon P3A was not expressed on cell surface of transfected HEK293 cells and introduction of αP3A23′G>A did not rescue the cell surface expression (Fig. 1e). Because an exhaustive search for other mutations in the α subunit, including single allele analysis by the ‘conversion' method^26^, detected no additional mutation, we examined the effects of αP3A23′G>A on pre-mRNA splicing.

### αP3A23′G>A markedly enhances inclusion of exon P3A in muscle

Allele-specific RT-PCR of biopsied muscles revealed that αP3A23′G>A mutation markedly enhanced incorporation of exon P3A into mature mRNA and prevented expression of the functional P3A(−) transcript (Fig. 1c). Allele-specific real-time RT-PCR of muscle mRNA similarly showed that αP3A23′G>A markedly reduced the functional P3A(−) transcript, whereas an allele harboring αG421R generated the P3A(−) transcript to similar levels as that of normal controls (Fig. 1d).

### αP3A23′G>A disrupts a putative exonic splicing silencer

**Figure 2:**
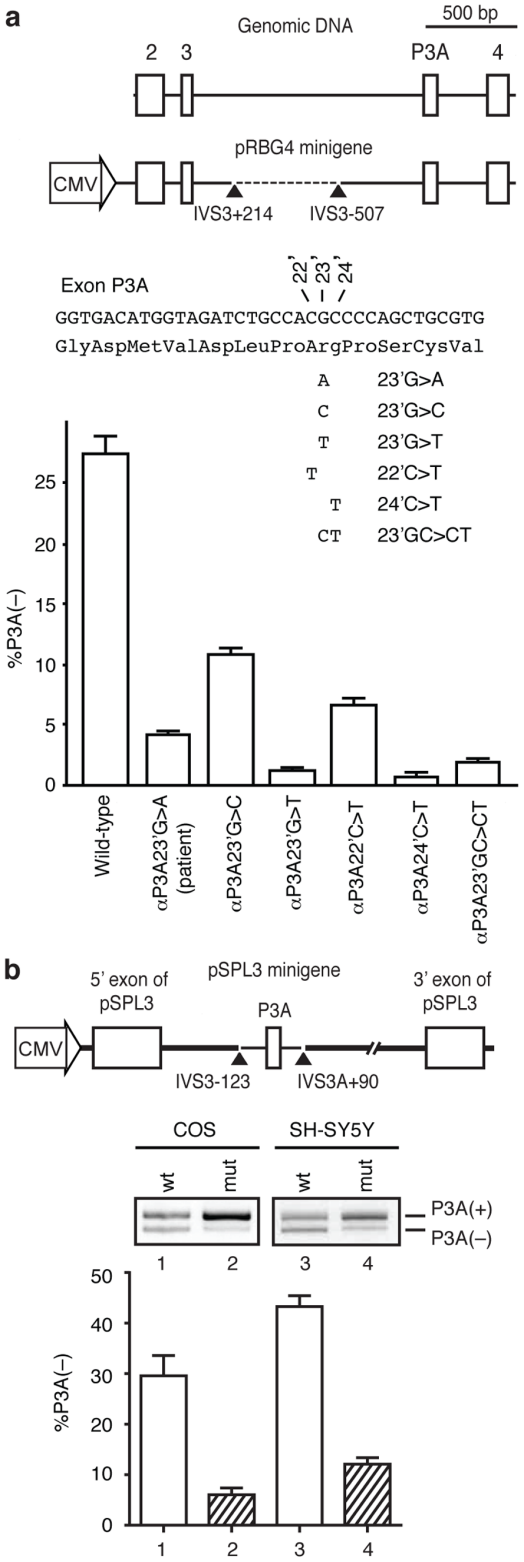
Construction of minigenes and splicing assays. (a) Structure of *CHRNA1* gene and pRBG4 minigene. A 589-bp segment (broken line) in intron 3 is deleted in pRBG4 minigene. The patient's mutation and five artificial mutations are introduced into pRBG4 minigene. Ratios of exon P3A skipping are quantified by real-time RT-PCR of COS cells transfected with pRBG4 minigenes. (b) pSPL3 minigene harboring *CHRNA1* exon P3A and flanking introns. Arrowheads point to the boundaries of *CHRNA1* and pSPL3 vector. Ratios of exon skipping are analyzed by RT-PCR of COS cells and SH-SY5Y cells transfected with pSPL3 minigenes. Bars and lines represent mean and standard deviation (SD), respectively, of three independent experiments for both (a) and (b).

We constructed a minigene harboring exons 2 to 4 of *CHRNA1* (Fig. 2a) in the pRBG4 mammalian expression vector to dissect the *cis*-element of splicing. We transfected COS cells with the wild-type and mutant minigenes and confirmed that the minigenes recapitulated the effect of the identified mutation on splicing (Fig. 2a). To examine whether the identified mutation disrupts an ESS or generates an ESE, we introduced five artificial mutations between nucleotide positions 22 and 24 (Fig. 2a). All mutants enhanced incorporation of exon P3A, indicating that G at position 23 as well as its flanking nucleotides constitute an ESS and αP3A23′G>A mutation disrupts it.

We also inserted exon P3A and its flanking introns between the two proprietary constitutive exons of the modified exon-trapping vector pSPL3 (Fig. 2b) to test whether the mutation can recapitulate the aberrant splicing in a heterologous context. The αP3A23′G>A in pSPL3 indeed reiterated the enhanced recognition of exon P3A in COS and SH-SY5Y cells (Fig. 2b), which suggested that exon P3A and its flanking intronic sequences are sufficient to regulate inclusion or skipping of exon P3A. We also compared splicing efficiencies of the wild-type P3A construct in pSPL3 in COS, SH-SY5Y, HEK293, and HeLa cells (Fig. 2b, and data not shown), and found that the P3A(−) and P3A(+) transcripts were expressed at a 1∶1 ratio in SH-SY5Y cells, as in human skeletal muscle^12^. We thus obtained a faithful splicing system with SH-SY5Y cells and pSPL3 constructs for further mechanistic analyses.

### αP3A23′G>A disrupts binding of hnRNP L while gains binding of hnRNP LL

**Figure 3:**
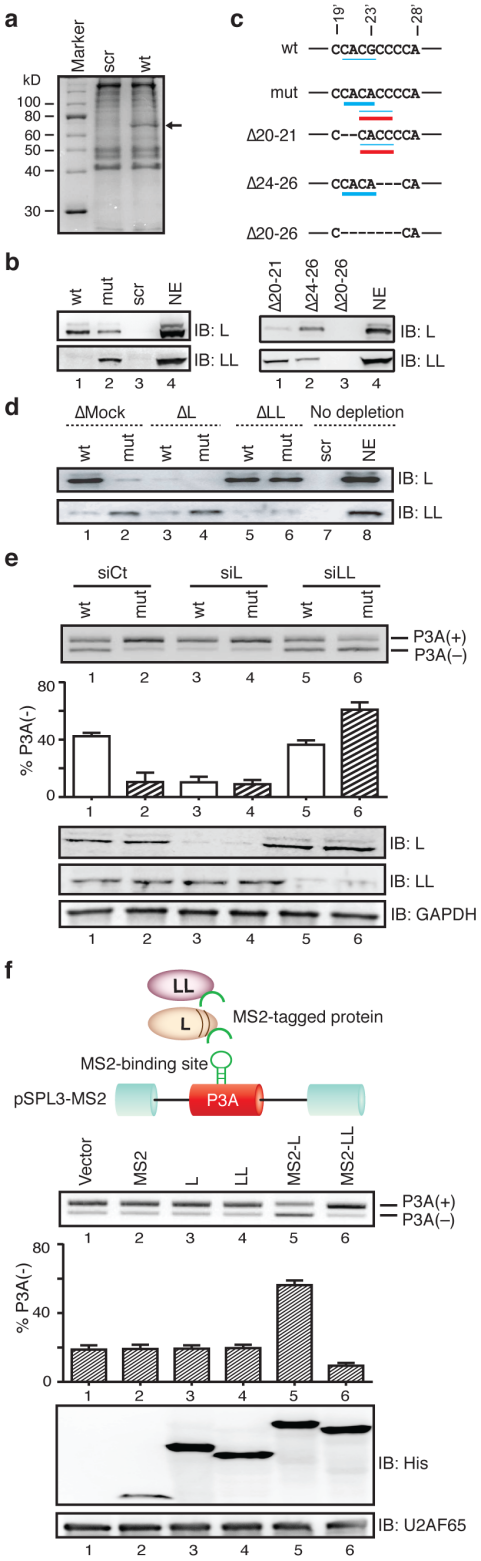
αP3A23′G>A compromises binding affinity for a splicing-suppressing hnRNP L and gains binding affinity for a splicing-enhancing hnRNP LL. (a) Coomassie blue staining of RNA affinity-purified products using SH-SY5Y nuclear extract with biotinylated RNA probes. A ∼70-kDa protein (arrow) is detected only with the wild-type (wt) probe but not with the scrambled (scr) probe. (b) Immunoblots (IB) probed with candidate splicing *trans*-factors. The wild-type exon P3A (wt) binds to hnRNP L (L) and the αP3A23′G>A (mut) disrupts its binding. The mutation gains *de novo* binding to hnRNP LL (LL). NE indicates 10% of nuclear extract used. (c) Deletion mutagenesis to map the binding sequences of hnRNPs L and LL in exon P3A. Underlines indicate putative binding motifs for hnRNP LL (red lines) and hnRNP L (thin and thick blue lines for weak and strong motifs, respectively)^27^. Note that the G-to-A mutation *de novo* generates a binding motif of CACC for hnRNPs L and LL. Immunoblots of RNA affinity-purified products are detected with antibodies against hnRNPs L and LL. (d) Mock-, hnRNP L-, and hnRNP LL-depleted SH-SY5Y nuclear extracts were affinity-purified with RNA probes and resolved by immunoblotting. (e) RT-PCR of wild-type (wt) and mutant (mut) pSPL3 minigenes in SH-SY5Y cells treated with siRNA against control (siCt), hnRNP L (siL), and hnRNP LL (siLL). Immunoblotting shows down regulation of hnRNPs L and LL. (f) Schematic presentation of a reporter minigene (pSPL3-MS2) and *trans*-acting effectors. HnRNPs L and LL (ovals) are fused to the artificial MS2 coat protein (inverted U shape). MS2 coat protein-binding hairpin RNA is substituted for the splicing regulatory site of exon P3A to directly tether MS2 coat protein-fused hnRNPs L and LL. RT-PCR of pSPL3-MS2 minigenes in SH-SY5Y cells that are co-transfected with the indicated effectors. Immunoblotting shows expression of effectors in the nuclear lysate of SH-SY5Y cells. The MS2-L construct has 16 additional codons arising from the multiple cloning site compared to MS2-LL. Bars and lines represent mean and SD, respectively, of three independent experiments for panels (e) and (f).

Having identified the critical *cis*-element of splicing, we next sought for a *trans*-factor responsible for regulating inclusion or skipping of exon P3A. RNA affinity purification of the nuclear extract prepared from SH-SY5Y cells with the wild-type P3A RNA probe yielded a ∼70 kDa protein (Fig. 3a). Analysis of the excised band by mass spectrometry revealed 26 unique peptides that matched to hnRNP L (the Mascot score of 343; significance threshold, *p* < 0.05). Immunoblotting demonstrated that the protein bound to the wild-type P3A exon was indeed hnRNP L with a predicted molecular weight of ∼68 kDa (Fig. 3b, lane1). As expected, the αP3A23′G>A-mutated probe diminished the binding affinity for hnRNP L (Fig. 3b, lane 2). We further examined binding of other candidate splicing factors; hnRNP LL, hnRNP K, hnRNP J, SRSF1 (formerly SF2/ASF), SRSF2 (formerly SC35), SRSF5 (formerly SRp40), and SRSF6 (formerly SRp55) (Fig. 3b and Fig. S2b). We found that none bound to the wild-type probe, but unexpectedly the mutation *de novo* gained binding for hnRNP LL (Fig. 3b, lane 2). We also resolved the RNA affinity-purified proteins bound to the mutant probe by mass spectrometry and identified hnRNP LL with the Mascot score of 124 (significance threshold, *p* < 0.05).

### Overlapping binding motifs are responsible for a competitive binding of hnRNP L and hnRNP LL

Previous reports suggest that both hnRNPs L and LL preferentially bind to CA-repeat or C/A-rich sequences^27^,^28^,^29^. *In vitro* SELEX studies of hnRNP L demonstrated that CACA and ACAC sequences confer high-affinity and that CACG and CACC sequences confer low-affinity binding motifs for hnRNP L^27^. Although no SELEX data are available for hnRNP LL, hnRNP LL prefers to bind to CACC sequence of the *CD45* transcript^28^,^29^. The wild-type exon P3A carries the low-affinity binding CACG site for hnRNP L (Fig. 3c). The αP3A23′G>A mutation rather changes the low-affinity CACG site to a high-affinity CACA site. In addition, this mutation acquires a low-affinity CACC site for hnRNP L, which also serves as a *de novo* binding site for hnRNP LL. Accordingly, this mutation disrupts the native CACG motif for hnRNP L and introduces two novel overlapping CACA and CACC motifs for hnRNP L as well as a novel CACC motif for hnRNP LL. Affinity-purification experiments, however, showed that the mutation abolishes binding of hnRNP L. We thus dissected the molecular basis of the loss of hnRNP L-binding.

Deletion of nucleotides 20 and 21 (Δ20–21) from the mutant sequence abrogates the high affinity CACA motif for hnRNP L but retains the CACC motif for hnRNPs L and LL (Fig. 3c). Affinity purification of nuclear extract from SH-SY5Y cells with Δ20–21 probe showed loss of hnRNP L and gain of hnRNP LL binding (Fig. 3c, lane 1). Similarly, deletion of nucleotides 24 to 26 (Δ24–26) from the mutant sequence generates a high affinity CACACA motif for hnRNP L, but abrogates the CACC motif for hnRNP LL. As predicted, Δ24–26 probe gave rise to binding of hnRNP L (lane 2). Additionally, deletion of nucleotides 20 to 26 (Δ20–26) from the mutant sequence abrogates all affinity motifs for hnRNPs L and LL, and indeed it did not bind to either hnRNP L or LL (lane 3). This suggests that hnRNPs L and LL compete for binding to the overlapping site of the mutated sequence in exon P3A, and consequently hnRNP LL wins the competition.

To further confirm the competitive binding of hnRNPs L and LL, we depleted hnRNP L or LL from nuclear extract of SH-SY5Y cells (Fig. S2c) and performed RNA affinity purification assays. As we predicted, depletion of hnRNP LL restored binding of hnRNP L to the mutant probe (Fig. 3d, lane 6), which underscored a notion that hnRNP LL competes with hnRNP L for binding to the mutant probe.

### HnRNP L silences and hnRNP LL enhances inclusion of exon P3A

We next examined the effects of hnRNPs L and LL on splicing of exon P3A by siRNA-mediated downregulation of hnRNPs L and LL in SH-SY5Y cells (Fig. 3e). Downregulation of hnRNP L induced inclusion of exon P3A in the wild-type minigene (Fig. 3e, lane 3), whereas downregulation of hnRNP LL caused skipping of exon P3A in the mutant minigene (lane 6), indicating that hnRNPs L and LL function as splicing silencer and enhancer, respectively. Expression of siRNA-resistant hnRNPs L (si-res L) and LL (si-res LL) along with siRNA in SH-SY5Y cells negated possible off-target effects of siRNAs (Fig. S3a).

We next confirmed that hnRNPs L and LL indeed work on the identified *cis*-element and not on the other sites. To this end, we tethered hnRNPs L and LL to the target using the bacteriophage MS2 coat protein. We prepared an effector construct expressing MS2-tagged hnRNP L or LL protein (MS2-L and MS2-LL, respectively), and the target minigene construct (pSPL3-MS2) containing the MS2-binding site, which was substituted for the native target site. As we expected, tethering of hnRNP L to the target promoted skipping of exon P3A (Fig. 3f, lane 5), whereas tethering of hnRNP LL induced inclusion of exon P3A (lane 6). Lack of splicing modulating effects of hnRNPs L and LL without MS2-tag indicates that neither hnRNP L nor LL works on the other sites (Fig. 3f, lanes 3 and 4). We also confirmed that MS2-fused hnRNPs L and LL had no effect on a minigene lacking the MS2-binding site (pSPL3-nonMS2) (Fig. S3b). Thus, hnRNP L and hnRNP LL exert silencing and enhancing activities on the identified target site.

### The proline-rich region of hnRNP L is essential for skipping of exon P3A

**Figure 4:**
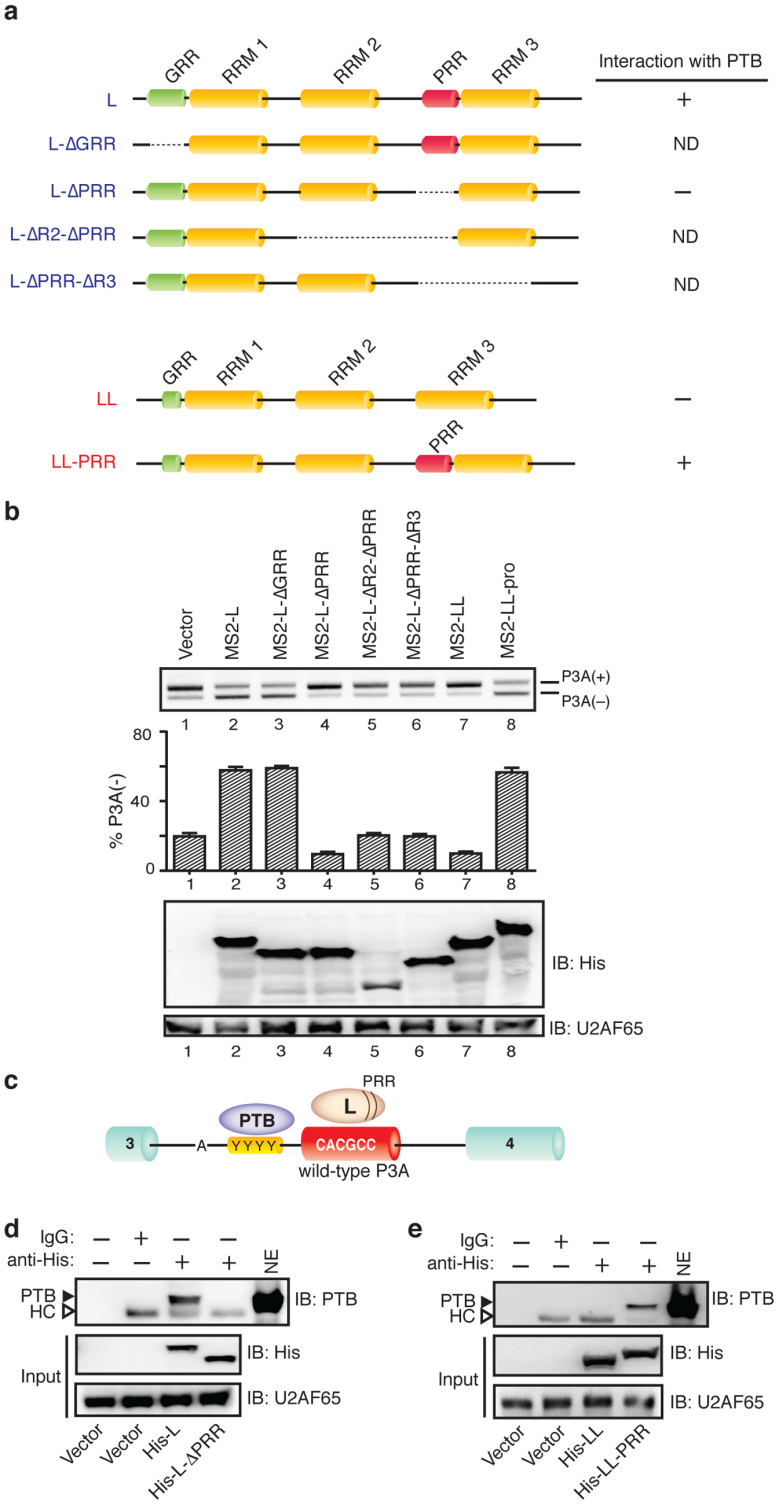
HnRNP L interacts with PTB through proline-rich region (PRR) to synergistically repress inclusion of exon P3A. (a) Schematic domain-structures of hnRNPs L and LL and their mutant derivatives. Interaction of each protein product with PTB is indicated on right according to the results from panels (d) and (e). RRM, GRR and ND indicate RNA recognition motif, glycine-rich region and not detected respectively. (b) RT-PCR of pSPL3-MS2 products after the indicated MS2-tagged *trans*-acting effectors are introduced into SH-SY5Y cells. Bars and lines represent mean and SD, respectively, of three independent experiments. Immunoblotting shows expression of effectors in the nuclear lysate of SH-SY5Y cells. (c) Schematic binding sites of hnRNP L and PTB in exon P3A and upstream PPT (YYYY) respectively. (d) Interaction of PTB with His-tagged hnRNP L and its indicated mutant. Nuclear extract of transfected SH-SY5Y cells is immunoprecipitated with anti-His antibody and assayed for interaction with endogenous PTB by immunoblotting (IB). Open arrowheads point to IgG heavy chain (HC) that was non-specifically precipitated. (e) Interaction of PTB with His-tagged hnRNP LL and its indicated mutant. See (d) for the procedures and other labels.

We next dissected functional domains of hnRNPs L and LL that dictate skipping and inclusion of exon P3A, respectively. HnRNP L (589 amino acids; accession number, NP_001524) and hnRNP LL (542 amino acids; NP_612403) are paralogues^29^ of similar size. They share an overall amino acid identity of 58%, and contain three highly conserved RNA-recognition motifs (RRMs) (Fig. 4a)^17^,^29^. HnRNP LL, however, differs in two domains from hnRNP L: its N-terminal glycine-rich region (GRR) is less prominent, and it lacks the proline-rich region (PRR) (Fig. 4a). We thus postulated that one or both of these distinct regions determine the splicing suppressing activity of hnRNP L. To prove this, we constructed a series of deletion mutants of hnRNP L and introduced them into SH-SY5Y cells along with the target pSPL3-MS2 substrate carrying the MS2-binding site. To prevent the possible effect of each deletion on a nuclear-localization signal, we introduced the nuclear-localization signal sequence of the SV40 large T-antigen at the N-terminal end of each construct. We found that deletion of GRR from the MS2-hnRNP L fusion construct (MS2-L-ΔGRR) had no effect on skipping of P3A exon (Fig. 4b, lane 3), whereas deletion of PRR (MS2-L-ΔPRR) caused inclusion of P3A exon (lane 4). Further deletions including RRM2 (MS2-L-ΔR2-ΔPRR) and RRM3 (MS2-L-ΔPRR-ΔR3) completely lost the splicing effects of hnRNP L (Fig. 4b, lanes 5 and 6). In contrast, artificial insertion of PRR into hnRNP LL (MS2-LL-PRR) transformed the exon P3A inclusion activity of hnRNP LL to the exon P3A skipping activity (Fig. 4b, lanes 7 and 8). We conclude that PRR is responsible for the exon skipping activity of hnRNP L, and the exon inclusion activity of hnRNP LL is attributed to the lack of PRR.

### HnRNP L and PTB cooperatively prevent inclusion of exon P3A

We next asked how PRR of hnRNP L confers exon skipping activity. We previously reported that PTB binds to polypyrimidine tract (PPT) of intron 3 and suppresses inclusion of exon P3A (Fig. 4c)^16^. Although direct binding of hnRNP L and PTB has been previously reported^30^, its mechanistic basis on splicing was not yet resolved. Based on our data, we hypothesized that PRR of hnRNP L binds to PTB and cooperatively suppresses inclusion of exon P3A. We thus examined the role of PRR of hnRNP L on the interaction with PTB. Histidine-tagged hnRNP L (His-L), its PRR-deleted variant (His-L-ΔPRR), histidine-tagged hnRNP LL (His-LL), and its PRR-inserted variant (His-LL-PRR) were expressed in SH-SY5Y cells, and they were immunoprecipitated with anti-histidine antibody in the presence of RNase. Immunoblotting revealed that PTB was precipitated with His-L but not with His-L-ΔPRR (Fig. 4d). On the other hand, PTB was not precipitated with hnRNP LL (His-LL) but was detected with His-LL-PRR (Fig. 4e). These results indicate that PRR of hnRNP L enables hnRNP L to interact with PTB.

We next examined whether the splicing suppressive effects of PTB and hnRNP L were additive or cooperative. Enhanced inclusion of exon P3A by knocking down of both hnRNP L (siL) and PTB (siPTB) was similar to that observed by siL or siPTB alone (Fig. S4), which suggested that PTB and hnRNP L cooperatively drive skipping of exon P3A with no additive effect.

### HnRNP L–PTB interaction impairs the exon-definition E complex formation to promote skipping of exon P3A

**Figure 5:**
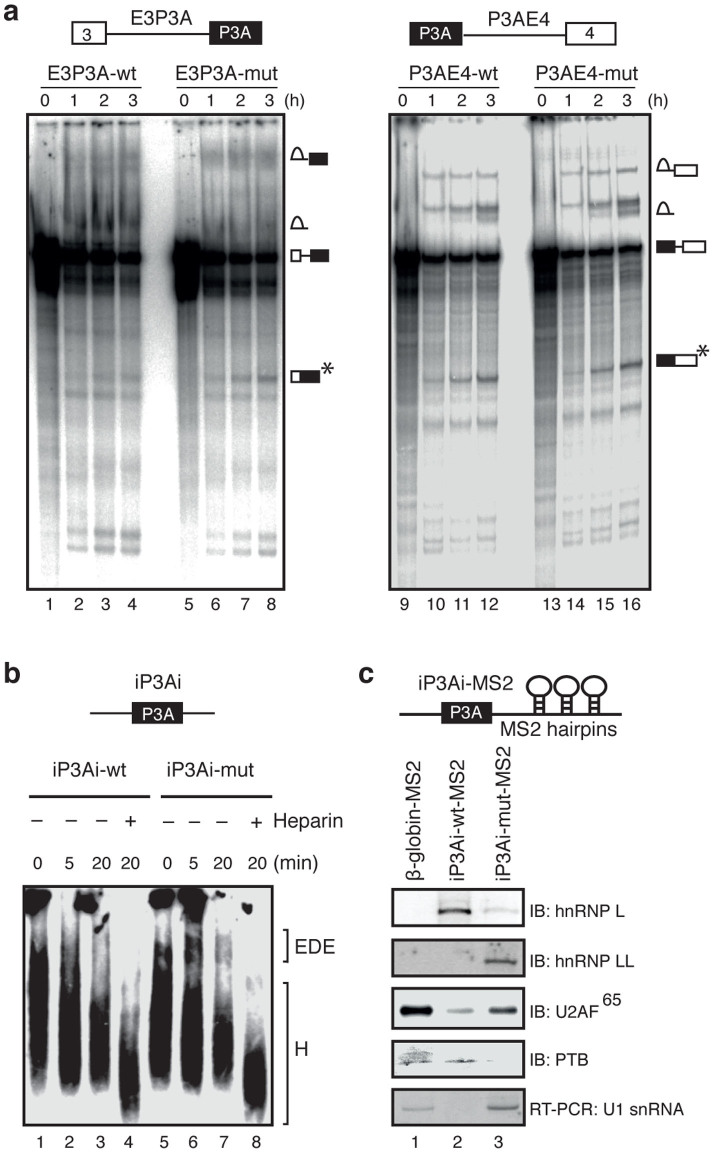
Skipping of exon P3A is promoted by impairing the formation of exon-defined E (EDE) complex in the wild-type pre-mRNA. (a) Time-course data obtained from *in vitro* splicing of the ^32^P-labeled pre-mRNAs from E3P3A (wt and mut) and P3AE4 (wt and mut) minigenes. The splicing products are shown schematically on the right. The spliced mRNA (asterisk) is increased in E3P3A-mut compared to E3P3A-wt. Although intron lariats are apparently increased in E3P3A-wt, the intron lariat and high molecular weight RNAs are not clearly resolved for E3P3A, and the increase of the intron lariats cannot be precisely estimated. Poor resolution of splicing products of E3P3A compared to P3AE4 is likely due to binding of a splicing repressing PTB. (b) Time-course analysis of early exon-defined spliceosome (EDE complex) that assembles across the P3A exon of ^32^P-labeled substrates (iP3Ai-wt and iP3A-mut) in the absence of ATP. Native agarose gel electrophoresis resolves the indicated nonspecific complex H (H) and the exon-defined E complex (EDE). (c) Schematic structures of MS2-attached wild-type (wt) and mutant (mut) substrates used for isolation of EDE complex. Immunoblotting (IB) and RT-PCR analyses of purified E complex assembled on indicated substrates. PTB was likely bound to a CUCUCUCU sequence in intron 1 of β-globin-MS2 pre-mRNA.

To further delineate the precise mechanism by which hnRNP L-PTB interaction causes skipping of exon P3A, we employed an *in vitro* splicing assay using a HeLa cell nuclear extract. Since PTB binds to PPT upstream of exon P3A and hnRNP L binds to exon P3A, we made two sets of splicing substrates, both of which carried either wild-type (wt) or mutant (mut) sequence. The structure of one set was “exon 3-intron 3-exon P3A” (E3P3A-wt and E3P3A-mut), and that of the other was “exon P3A-intron P3A-exon 4” (P3AE4-wt and P3AE4-mut). This substrate system could inform us on whether the hnRNP L-PTB interaction affects the 3′ or 5′ splice site of exon P3A. E3P3A-mut was spliced more efficiently than E3P3A-wt, whereas P3AE4-mut was spliced as efficiently as P3AE4-wt (Fig. 5a). These results indicate that the binding of hnRNP L to wild-type exon P3A suppresses removal of the upstream intron but not of the downstream intron.

We next analyzed the early ATP-independent complex across the exon, previously termed the exon-defined E (EDE) complex^31^,^32^, with an RNA substrate comprised of exon P3A and the flanking introns (iP3Ai; Fig. 5b). We isolated the EDE complex using MS2-attached iP3Ai RNA substrate, and analyzed the associated factors by immunoblotting and RT-PCR. We confirmed the binding of hnRNP L to the iP3Ai-wt-MS2 probe, and hnRNP LL binding to the iP3Ai-mut-MS2 probe (Fig. 5c, lanes 2 and 3). We found that iP3Ai-mut-MS2 associated with U2AF^65^ (lane 3), whereas the association of U2AF^65^ with iP3Ai-wt-MS2 was less efficient (lane 2). In contrast, iP3Ai-wt-MS2 associated with PTB (lane 2), whereas iP3Ai-mut-MS2 could not associate with PTB (lane 3). We also analyzed associated U1 snRNA by RT-PCR. We detected U1 snRNA with iP3Ai-mut-MS2 and control β-globin-MS2 (Fig. 5c, lanes 1, 3) but not with iP3Ai-wt-MS2 (lane 2).

**Figure 6:**
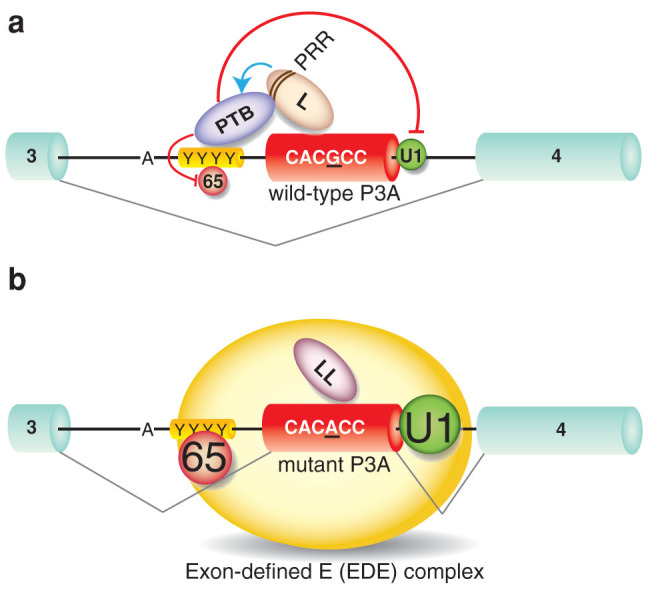
Model of pathogenic mutation (αP3A23′G>A)-induced aberrant exon P3A inclusion that is antagonistically regulated by hnRNPs L and LL. Early spliceosome complex formation on *CHRNA1* pre-mRNA with alternative exon P3A are schematically shown. Large letters indicate functional binding of splicing factors, whereas small letters represent compromised binding of splicing factors. The sequence of point mutation in exon P3A (αP3A23′G>A) is underlined. (a) HnRNP L (L) binds to wild-type exon P3A and interacts with PTB through the proline-rich region (PRR), which stabilizes PTB binding to the upstream PPT (YYYY). The hnRNP L–PTB interaction prevents association of U2AF^65^ (65) to PPT and U1 snRNP (U1) to the 5′ splice site. The formation of exon-defined E (EDE) complex is thus impaired, which leads to skipping of exon P3A. (b) The αP3A23′G>A-mutation switches binding of hnRNP L to hnRNP LL (LL). Lack of PRR in hnRNP LL fails to stabilize PTB binding to the upstream PPT, which allows binding of U1 snRNP (U1) and U2AF^65^ (65) on pre-mRNA. The formation of the exon-defined E (EDE) complex facilitates inclusion of exon P3A.

Taken together, our findings indicate that the switching mechanism of exon P3A skipping and inclusion is triggered by hnRNP L and hnRNP LL, respectively. In the wild-type *CHRNA1* pre-mRNA, the binding of hnRNP L to the target sequence in exon P3A stabilizes association of PTB to the upstream PPT. The stabilized association precludes binding of U2AF^65^ to the PPT and also binding of U1 snRNP to the downstream 5′ splice site. The impaired definition of exon P3A thus gives rise to exon P3A-skipped mRNA (Fig. 6a). In the mutant *CHRNA1* pre-mRNA, the binding of hnRNP LL to the mutant sequence in exon P3A excludes the competing hnRNP L, allowing association of U2AF^65^ and U1 snRNP to the upstream PPT and downstream 5′ splice site, respectively; this, in turn, leads to the exon P3A definition that favors formation of the exon P3A-included species of mRNA (Fig. 6b).

## Discussion

Exonic and intronic mutations that affect *cis*-acting splicing elements have been reported in many diseases^2^,^3^. More than 16–20% of missense mutations of the human mismatch-repair genes *hMSH2* and *hMLH1* are predicted to disrupt exonic splicing enhancers, which modulate splicing of transcripts containing mutated exons^33^. However, splicing mutations in nonfunctional exons have not been well studied because they are often considered rare polymorphisms. We here identified a pathogenic splicing mutation (αP3A23′G>A) in a nonfunctional exon P3A of *CHRNA1* in a CMS patient. Thus our study underscores the importance of including nonfunctional exons in mutation analysis.

We confirmed exclusive inclusion of exon P3A in the patient's muscle. Due to lack of available human skeletal muscle cell lines, we used the SH-SY5Y human neuroblastoma cell line because the splicing patterns of our minigenes in SH-SY5Y cells were similar to those in the patient. HnRNP L is expressed ubiquitously and abundantly in almost all cell types, whereas prominent expression of hnRNP LL has been reported only in lymphoid cells, activated T-cells, and testes^28^. The EST profile in the NCBI UniGene database shows that hnRNPs L and LL are similarly expressed in human skeletal muscle. The equivalent expression levels of hnRNPs L and LL in SH-SY5Y cells enabled us to recapitulate the splicing patterns we observed in patient muscle.

In *CHRNA1* exon P3A, the mutation still retains suboptimal binding motifs of hnRNP L but hnRNP LL competitively prevents binding of hnRNP L. Specific binding motifs of hnRNP L on *CD45* exons 4, 5, and 6^19^,^28^,^34^ and those of hnRNP LL on *CD45* exons 4 and 6^28^,^29^ have previously been analyzed. In contrast to the event in *CHRNA1*, hnRNPs L and LL bind noncompetitively to adjacent sites within a single silencer element on *CD45* exon 4 and suppress splicing cooperatively. Competitive binding of antagonizing splicing *trans*-factors to an identical exon has been observed in *SMN1* and *SMN2* pre-mRNAs. *SMN1* and *SMN2* genes are highly homologous paralogues differing only at 6th nucleotide of exon 7 in T vs C, respectively. Inclusion of *SMN1* exon 7 is enhanced by SRSF1. The T-to-C substitution in *SMN2* abolishes binding of splicing-stimulating SRSF1^35^,^36^, and enhances binding of a splicing-suppressing hnRNP A1^37^,^38^. In contrast to hnRNPs L and LL in the case of *CHRNA1* pre-mRNA, SRSF1 and hnRNP A1 do not compete for binding to the overlapping target site. Competitive binding of two antagonizing splicing *trans*-factors to the same target is thus unique to *CHRNA1* exon P3A.

The splicing repressor activity of PTB has been extensively characterized^39^,^40^,^41^,^42^. Splicing repressor activity of hnRNP L has been extensively investigated in *CD45* pre-mRNA^18^,^43^,^44^. We here report a novel mechanism of PTB-mediated inhibition of exon-defined spliceosome formation, in which hnRNP L facilitates binding of PTB to the upstream PPT that suppresses subsequent association of U2AF^65^ and U1 snRNP in the exon-defined E complex. Another example of PTB-hnRNP association has been reported for *PKM* encoding pyruvate-kinase-M, where PTB and hnRNPs A1/A2 cooperate in excluding exon 9 to increase lactate production in cancer cells^45^. Together, hnRNP proteins appear to be functional partners of PTB which binds to upstream PPT to inhibit E complex formation and leads to the subsequent splicing suppression.

We have identified unique PRR in hnRNP L that plays an essential role in binding of PTB to PPT to suppress splicing activity. Interestingly, a recent report showed that a PRR-containing linker domain of hnRNP L binds to hnRNP A1 in an RNA-dependent manner and binding of both molecules to *CD45* exon 4 causes skipping of exon 4^46^. Similarly, a specific peptide motif and adjacent PRR of Raver1 are essential for PTB-mediated splicing repressor activity of *Tpm1* pre-mRNA^47^. In contrast to hnRNP L, however, the PRR of Raver1 is not necessary for binding to PTB.

In contrast to hnRNP L, the molecular mechanisms of splicing-modulating activity of hnRNP LL has not been extensively elucidated. A recent study reported the variations of domain structure between hnRNPs L and LL, which exhibit functional alterations^48^. Here, we first prove that lack of PRR accounts for lack of interaction of hnRNP LL with PTB, which destabilizes PTB-binding to the upstream PPT. This, however, is unlikely to be an exclusive splicing-enhancing mechanism of hnRNP LL, because tethering of hnRNP LL to exon P3A decreased the ratio of exon-skipped transcript further than a null-tethered control (from 18.4% to 9.8% in Fig. 3f, lanes 1 and 6). Although the effect of hnRNP LL on *CHRNA1* exon P3A is not as conspicuous as that of hnRNP L, hnRNP LL is likely to have a yet unidentified positive stimulatory effect on splicing.

An important question is why humans and great apes acquired alternative splicing of *CHRNA1* transcripts in the course of evolution. Alternative exons have evolved by exonization of retroposed mobile elements, whereby new exons are generated following changes in noncoding regions of a gene^10^,^49^. Exon P3A and its flanking intronic region have indeed arisen from exonization of the retroposed mammalian interspersed repeat element (MIR)^50^. Unlike exonizations of *Alu* repeats, which is common in primates^49^, exonizations of MIRs are mostly ancient events that occurred ∼130 million years ago in mammals^10^. As only human and great apes carry exon P3A and because no homologous sequence is present in other mammals, exonization of MIR leading to the generation of exon P3A is likely a relatively recent event. Acquisition of exon P3A is predicted to be detrimental for the neuromuscular signal transmission. Human and great apes, however, acquired intelligence, which was likely more important than muscle strength for survival and reproduction. Alternatively, PTB and hnRNP L are dominantly expressed at nuclei close to the NMJ to generate the functional P3A(−) transcript. In mammals, *CHRNE* encoding the AChR ε subunit is expressed only at the endplate to achieve endplate-specific expression of AChR^51^,^52^. Alternative splicing of *CHRNA1* pre-mRNA to include or skip exon P3A might have evolved as an additional means to achieve the endplate-specific expression of AChR.

## Methods

### Muscle biopsies, endplate studies, and mutation analysis

All human studies including the experimental protocols were approved by the Institutional Review Board of Mayo Clinic and the Ethical Review Committee of Nagoya University Graduate School of Medicine. An appropriate informed consents were obtained from all human subjects investigated in this study at Mayo Clinic. Intercostal muscle specimens were obtained intact from origin to insertion from the patient and control subjects without muscle disease undergoing thoracic surgery. AChR and acetylcholinesterase were detected in cryostat sections by two-color fluorescence^53^. EPs were localized for electron microscopy and analyzed by established methods^54^,^55^. Peroxidase-labeled α-bungarotoxin was used for the ultrastructural localization of AChR^56^. Miniature EP potential (MEPP), miniature EP current (MEPC), and EP potential (EPP) recordings were performed, and estimates of the number of transmitter quanta released by nerve impulse were obtained as previously described^54^,^57^. We directly sequenced AChR α, β, δ, and ε subunit genes using genomic DNA isolated from muscle as previously described^58^. Genomic DNA from single allele was extracted from leucocytes using the ‘conversion' method^26^ by GMP Genetics.

### Expression of AChRs on HEK293 cells

The human α, β, δ, and ε subunit cDNAs were introduced into HEK293 cells and the total number of [^125^I]α-bungarotoxin binding sites expressed on cell surface was determined as previously described^58^.

### Construction of pRBG4 and pSPL3 minigenes for splicing analysis

We constructed pRBG4 minigene spanning exons 2 to 4 of *CHRNA1* to analyze pre-mRNA splicing. We also constructed another pSPL3 minigene, spanning exon P3A and flanking intronic sequences in the modified exon-trapping vector, pSPL3 (a discontinued product of Invitrogen). Naturally occurring and artificial mutations were engineered into the pRBG4 and pSPL3 minigenes using the QuikChange Site-Directed Mutagenesis Kit. The absence of artifacts was confirmed by sequencing the entire inserts.

### RT-PCR for splicing analysis

Total RNA was extracted 40 h after transfection using Trizol (Invitrogen) or the RNeasy Mini kit (Qiagen), followed by DNase I treatment. cDNA was synthesized with an oligo-dT primer using Superscript II reverse transcriptase (Invitrogen) or ReverTra Ace (Toyobo). The ratio of the P3A(−) transcript to the total *CHRNA1* transcripts was calculated using the following equation: 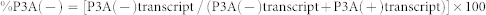


### RNA affinity purification assay and mass spectrometry

Biotinylated RNAs were synthesized *in vitro*, and RNA affinity purification assay was performed with a nuclear extract of SH-SY5Y cells as previously^15^. The purified proteins were fractionated on a 10% SDS-polyacrylamide gel and stained with Coomassie blue or analyzed by immunoblotting as we previously described^15^. A Coomassie blue-stained band was excised from the gel and was digested in-gel by Trypsin Gold (Promega) according to the manufacturer's protocols. For in-solution digestion, the RNA-bound proteins were eluted in elution buffer (0.1 M glycine with 2 M urea, pH 2.9) and digested by Trypsin Gold according to the manufacturer's recommendations. Nanoelectrospray tandem mass analysis was performed using an LCQ Advantage Mass Spectrometry System (Thermo Finnigan). Multiple MS/MS spectra were analyzed by the Mascot program version 2.4.1 (Matrix Science).

### Depletion of hnRNP L and hnRNP LL from nuclear extract

Antibody mediated depletion of hnRNP L and hnRNP LL from SH-SY5Y cell nuclear extract was performed using Protein G HP spin trap (GE Healthcare) according to the manufacturer's instructions.

### siRNA knockdown and minigene splicing

siRNAs were synthesized to downregulate hnRNP L, hnRNP LL, and PTB by Sigma Genosys: 5′-GAAUGGAGUUCAGGCGAUGTT-3′ for human hnRNP L^17^, 5′-AGUGCAACGUAUUGUUAUATT-3′ for human hnRNP LL^17^ and 5′-GCCUCUUUAUUCUUUUCGGTT-3′ for human PTB^16^. The control siRNA was AllStar Negative Control siRNA (1027281) by Qiagen. For the siRNA rescue assay, we purchased the human cDNA clones for *HNRNPL* (clone ID 6174088) and *HNRPLL* (clone ID 3502860) from Open Biosystems. We cloned each cDNA in pcDNA3.1/V5-His TOPO and introduced four silent mutations into the siRNA target region of each of hnRNPs L and LL using the QuikChange site-directed mutagenesis.

### Tethered function assay of hnRNP L and hnRNP LL

Tethered function assay was performed by co-transfection of a reporter minigene and an effector construct in which a particular RNA binding molecule was fused with the bacteriophage MS2 coat protein so that it binds to the artificially inserted target site in the reporter minigene. To construct a reporter minigene, we substituted the bacteriophage MS2 coat protein-binding hairpin RNA sequence (5′-ACATGAGGATCACCCATGT-3′) for the native sequence (5′-CACGCCC-3′) of exon P3A in *CHRNA1* (nucleotide numbers 20′–26′) in pSPL3 minigene using the QuikChange Site-Directed Mutagenesis Kit. We thus eliminated the binding sequence of both hnRNPs L and LL from exon P3A and introduced an artificial MS2 coat protein target site. We also constructed a pSPL3-nonMS2 minigene lacking the target site of MS2 coat protein. Effector constructs of hnRNPs L and LL were employed with or without the MS2 coat protein.

### Co-immunoprecipitation

Protein-protein interactions were studied by co-immunoprecipitation (Co-IP) experiment using the Nuclear Complex Co-IP kit (Active Motif) according to the manufacturer's instructions in the presence of RNase A (Ambion). We incubated 100 μg of nuclear extract with 2 μg of anti-His-tag antibody. We included two IP controls: one with normal IgG and the other without IgG. We increased the IP stringency by increasing the final concentration of NaCl up to 150 mM. We harvested bound molecules with protein G beads followed by boiling and analyzed them by immunoblotting using the antibody against PTB.

### Antibodies

Antibodies used in this study were anti-hnRNP L 4D11 (sc-32317, Santa Cruz Biotechnology), anti-hnRNP LL (Aviva System Biology), anti-hnRNP K/J (sc-32307, Santa Cruz Biotechnology, Inc.), anti-His-tag (D293-1, Medical & Biological Laboratories), anti-SF2/ASF (Zymed), anti-SR proteins 1H4 (sc-13509, Santa Cruz Biotechnology), anti-GAPDH (Sigma-Aldrich), anti-β-actin C4 (sc-47778, Santa Cruz Biotechnology), anti-PTB (sc-16547, Santa Cruz Biotechnology), and anti-U2AF^65^ MC3 (sc-53942, Santa Cruz Biotechnology).

### *In vitro* splicing and spliceosomal E complex assays

*In vitro* splicing was performed as described previously^32^ with minor modifications. ^32^P-labeled pre-mRNA (∼20 fmol) was incubated with 3.5 μl of HeLa cell nuclear extract (CilBiotech) for the indicated time at 30°C for P3AE4-wt/mut. As splicing efficiencies of E3P3A-wt/mut were poor at standard temperature of 30°C, we improved the efficiencies by pre-incubating the reaction mixture for 15 min at 37°C. The reaction mixture of 12.5 μl contained 3 mM ATP, 20 mM creatine phosphate, 20 mM HEPES-NaOH (pH 7.3), and 3.5 mM MgCl_2_. RNA was extracted with phenol, precipitated with ethanol, and fractionated by denaturing 7% or 10% PAGE. Spliceosomal E complex assay was performed as previously described^32^ except of the use of 1 × Tris-glycine and 2% low-melting-point agarose (Invitrogen) submarine gel electrophoresis at 4°C.

### MS2-affinity isolation of spliceosomal E complex of exon P3A

One pmol of the RNA probe (MS2-human-β-globin, MS2-iP3Ai-wt, or MS2-iP3Ai-mut) was incubated with 20-fold molar excess of MS2-MBP fusion protein^59^, prior to mixing with HeLa nuclear extract. Fifty μl of HeLa nuclear extract was preincubated with 10 μl (bead volume) of amylose resin (New England Biolabs) overnight at 4°C. The purified HeLa nuclear extract was incubated at 37°C for 30 min with a mixture of the RNA probe and the MS2-MBP fusion protein at final concentrations of 60 mM KCl and 25% of HeLa nuclear extract. Ten μl (bead volume) of amylose resin was added and rotated at 4°C for 30 min. The resin was washed four times with wash buffer (20 mM HEPES pH 8.0, 150 mM KCl, and 0.05% Triton X-100), and finally eluted with 10 mM maltose solution and subjected to SDS-PAGE and immunoblot analyses. To detect U1 snRNA, total RNA was purified from the indicated RNA-affinity-isolated spliceosomal E complex using TRI Reagent (Sigma-Aldrich) according to the manufacturer's instructions. After making cDNA with ReverTra Ace (Toyobo), U1 snRNA was detected using primers: 5′-GGGGAAGCTTCAGGGGAAAGCGCGAACGCAGTCC-3′ and 5′-GGGGGATCCATACTTACCTGGCAGGGGAGATACC-3′.

## Author Contributions

A.G.E. and Ki.O. conceived the project. M.A.R., A.M.,^1^ Ke.O. and M.I. designed experiments; M.A.R. performed most of the experiments; Ki.O., D.O.H., Ke.O. contributed to genetic studies, electrophysiological studies, and *in vitro* spliceosome studies, respectively. M.A.R., Ke.O., A.M.,^4^ A.G.E. and Ki.O. wrote the paper.

## Supplementary Material

Supplementary Information Supplementary Information

## Figures and Tables

**Table 1:** Morphometric and electrophysiological studies of endplates of the patient

	Patient	Controls
AChR index	0.87 ± 0.01 (27)	3.01 ± 0.11 (85)
EPP quantal content^a^	27 ± 8 (18)	31 ± 1
MEPP amplitude (mV)^b^	0.23 ± 0.015 (16)	1.00 ± 0.025 (165)

Values represent mean ± standard error (SE). Numbers in parenthesis indicate number of endplates (EPs).

^a^Quantal content of EP potential (EPP) at 1 Hz stimulation corrected for resting membrane potential of −80 mV, nonlinear summation, and non-Poisson release.

^b^The amplitude of the miniature EP potential (MEPPs) are corrected for resting membrane potential of −80 mV and a mean muscle fiber diameter of 5 μm. Temperature was 29 ± 0.5°C for EPP and MEPP recordings.
