# Supplementary material for: HnRNP L and hnRNP LL antagonistically modulate PTB-mediated splicing suppression of CHRNA1 pre-mRNA
Source: Sci Rep. 2013 Oct 14;3:2931. doi: 10.1038/srep02931 (PMC3796306; doi:10.1038/srep02931)
Supplement: Supplementary Information [file srep02931-s1.pdf]

Supplementary Information

HnRNP L and hnRNP LL antagonistically modulate PTB-mediated splicing suppression of *CHRNA1* pre-mRNA

Mohammad Alinoor Rahman[1], Akio Masuda[1], Kenji Ohe[1], Mikako Ito[1], David O. Hutchinson[2, 3], Akila Mayeda[4], Andrew G. Engel[2] and Kinji Ohno[1, 2]

[1]Division of Neurogenetics, Center for Neurological Diseases and Cancer, Nagoya University Graduate School of Medicine, Nagoya, Aichi, Japan
[2]Department of Neurology, Mayo Clinic, Rochester, Minnesota, U. S. A.
[3]Department of Neurology, Auckland City Hospital, Auckland, New Zealand
[4]Division of Gene Expression Mechanism, Institute for Comprehensive Medical Science (ICMS), Fujita Health University, Toyoake, Aichi, Japan

Address correspondence to: Kinji Ohno, Division of Neurogenetics, Center for Neurological Diseases and Cancer, Nagoya University Graduate School of Medicine,
65 Tsurumai, Showa-ku, Nagoya, Aichi 466-8550, Japan
Phone: +81-52-744-2446, Fax: +81-52-744-2449, e-mail: ohnok@med.nagoya-u.ac.jp

Supplementary information includes:

Supplementary Methods

Supplementary Figures S1-S4

Supplementary Methods

Allele-specific RT-PCR

The wild-type αG421 and mutant αR421 transcripts were discriminatively amplified using primers, 5'-CAAACACGGCTAGGGTaCC-3' and 5'-CAAACACGGCTAGGGTaCT-3', respectively, where the lower case 'a' representing an artificially introduced mismatched nucleotide to enhance discrimination between two alleles[1] and the underlined C and T are complementary to the wild-type and mutant nucleotides, respectively. The reverse primer in exon 2 was 5'-GTCCTGGGCTCCGAACAT-3'. The size of the expected allele-specific RT-PCR product was 1288 bp. The amplicon was then used as a template for nested RT-PCR using primers, 5'-AACCAATGTGCGTCTGAAAC-3' in exon 3 and 5'-TTTTTCACACCGCCATAGTC-3' in exon 4, which spanned exon P3A. With these nested primers, a transcript lacking exon P3A would yield a 78-bp fragment, whereas a transcript harboring exon P3A would generate a 153-bp fragment. Next, transcripts lacking and harboring exon P3A were specifically amplified using primers, 5'-AATGTGCGTCTGAAACAGCAA-3' and 5'-AATGTGCGTCTGAAACAGGGT-3', in which the underlined nucleotides anneal to the 5' end of exons 4 and P3A, respectively. The reverse primer was 5'-TCTCTGCTCTGGTAGGTTCC-3' in the 3' untranslated region, and the expected allele-specific RT-PCR product size was 1209 bp. The amplicon was used as a template for nested RT-PCR using primers, 5'-GGCTTCCACTCTCCCCTGAT-3' in exon 8 and 5'-TTCCAGGGCAGAGCTAAGC-3' in the 3' untranslated region, to amplify a 269-bp fragment that includes αG421R in exon 9. The nested RT-PCR product was then digested with NlaIV restriction enzyme (New England Biolab). A fragment carrying wild-type αG421 should be cleaved to produce 183- and 86-bp fragments, whereas a fragment harboring the mutant αR421 should remain uncut.

Construction and expression of wild-type and mutant AChRs

Sources of human α, β, δ, and ε subunit cDNAs were previously described[2-4]. We amplified the α subunit cDNA containing exon P3A from control skeletal muscle by RT-PCR. All cDNAs were cloned into the CMV-based expression vector pRBG4[5] for expression in human embryonic kidney (HEK293) fibroblasts. αP3A23'G>A and αG421R mutations were introduced into pRBG4 using the QuikChange site-directed mutagenesis kit (Stratagene), and the obtained constructs were verified by sequencing the entire inserts. Grown HEK293 cells on a 10-cm^2 dish were transfected with 5.4 μg of wild-type or mutant α subunit cDNA along with 2.7 μg each of

wild-type β, δ, and ε subunit cDNAs using 40.8 μl FuGENE-6 (Roche) according to the manufacturer's recommendations. To correct for cell numbers, transfection efficiency, and harvesting efficiency, we added 125 ng of the Renilla luciferase vector, phRL-TK (Promega), to each dish. The total number of [^{125}I]α-bungarotoxin binding sites expressed on surface of transfected HEK293 cells was determined as previously described[3]. Renilla luciferase activities were measured using the Renilla luciferase assay system (Promega) in a Turner Designs TD-20/20 Luminometer.

Construction of pRBG4 and pSPL3 minigenes for splicing analysis

To construct pRBG4 minigene, we first amplified a 500-bp genomic fragment spanning exon 2 (18th nucleotide) to intron 3 (214th nucleotide) by PCR using *Pfu* DNA polymerase (Stratagene) and control genomic DNA. The 5' ends of the forward and reverse primers carried XbaI and KpnI sites, respectively. Second, an 894-bp genomic fragment spanning intron 3 (507th nucleotide from the 3' end) to exon 4 (36th nucleotide) was similarly amplified by PCR. The 5' ends of the forward and reverse primers carried KpnI and ClaI sites, respectively. The two PCR fragments were ligated using the KpnI site and then subcloned into the XbaI and ClaI sites of the CMV-based expression vector pRBG4[5]. Compared to wild-type *CHRNA1*, the minigene lacked a 589-bp segment in the middle of intron 3 (Fig. 2a). Because nonsense-mediated mRNA decay can destroy a nonsense codon-containing transcript[6], we added the Kozak consensus sequence[7], 5'-CCACCATG-3', at the 5' end of the insert, to assure inframe translation of the minigene transcripts. The open reading frame in this minigene construct was the same as that of *CHRNA1.* A stop codon (TAA) should appear 11 codons downstream of the *CHRNA1* insert with this open reading frame.

We constructed pSPL3 minigene spanning exon P3A with flanking intronic sequences using the modified exon-trapping vector, pSPL3 (a discontinued product of Invitrogen), in which we replaced the original simian virus 40 (SV40) promoter with the CMV promoter and introduced NotI and PacI restriction sites in the intron[8]. We amplified a 288-bp genomic fragment spanning intron 3 (123th nucleotide from the 3' end) to intron P3A (90th nucleotide) by PCR. The PCR primers carried NotI and PacI sites at the 5' ends, respectively. The PCR fragment was subcloned into the NotI and PacI sites of the modified pSPL3 vector (Fig. 2b).

Cell culture and transfection

COS and HEK293 cells were cultured in the Dulbecco's minimum essential medium (DMEM, Sigma-Aldrich) supplemented with 10% fetal bovine serum (FBS, Sigma-Aldrich). SH-SY5Y

cells were cultured in Ham's F12 (DS Pharma Biomedical) and Eagle's minimal essential medium (EMEM, DS Pharma Biomedical) at a ratio of 1:1, which were supplemented with 2 mM L-glutamine, 1% non-essential amino acids (NEAA), and 15% FBS.

RT-PCR for splicing analysis

We estimated the absolute copy numbers of the P3A(–) and P3A(+) transcripts in muscles and in transfected cells with the LightCycler 1.2 real-time PCR instrument and the LightCycler FastStart DNA Master SYBR Green I kit (Roche) according to the manufacturer's recommendations. For specific amplification of the P3A(–) transcript, we used a forward primer 5'-AATGTGCGTCTGAAACAGCAA-3' at the exons 3/4 junction. As a reverse primer, we used 5'-TGTGAAATTTGTGATGCTATTG-3' at the polyadenylation signal site for pRBG4 minigenes and 5'-ACGTGATGTGGCCAGTGTACTG-3' on exon 5 for muscle specimens. For specific amplification of the P3A(+) transcript, we used primers 5'-ACCACCGCCAGGTCGTGG-3' on exon 2 and 5'-CTCATTCTGCAGATGAGAAAAC-3' on exon P3A. Control fragments amplified by the three primer pairs were cloned into pGEM-T (Promega) and used as templates for making standard curves of real-time RT-PCR. We also used the cloned control constructs to make sure that the three primer pairs did not cross-amplify the P3A(–) and P3A(+) transcripts.

Splicing efficiencies were estimated by conventional RT-PCR for pSPL3 minigenes using primers 5'-TCTGAGTCACCTGGACAACC-3' and 5'-ATCTCAGTGGTATTTGTGAGC-3' spanning the proprietary 5' and 3' exons of pSPL3. The intensities of scanned PCR-amplified spliced products were quantified with the ImageJ 1.44 software (NIH).

Harvesting cells for immunoblotting

Cells were harvested in PBS containing the Phosphatase Inhibitor Cocktail (Active Motif) by centrifugation at 2,000 x *g* for 5 min, and the pellets were resuspended in buffer A [10 mM HEPES-NaOH (pH 7.8), 10 mM KCl, 0.1 mM EDTA, 1 mM DTT, 2 mg/ml aprotinin, 0.5 mM PMSF, and 0.1% Nonidet P-40] with the Protease Inhibitor Cocktail and kept for 30 min on ice. After sonication, samples were centrifuged at 20,000 x *g* for 5 min and the supernatants were used as total cell lysate. For preparation of nuclear lysates, cells were kept for 30 min on ice in buffer A. Nuclei were pelleted and the supernatant containing cytoplasmic proteins were removed. The nuclei were then resuspended in buffer C [50 mM HEPES-NaOH (pH 7.8), 420 mM KCl, 0.1 mM EDTA, 5 mM $MgCl_2$, 2% glycerol, 1 mM DTT, 2 mg/ml aprotinin, and 0.5 mM PMSF] with the Protease Inhibitor Cocktail. After vortexing briefly and stirring for 30 min

at 4 ˚C, they were centrifuged at 20,000 x *g* for 5 min and the supernatants were used as nuclear lysate. Obtained lysates were analyzed by a 10% SDS polyacrylamide gel electrophoresis and immunoblotting was performed as previously described[9].

RNA affinity purification assay

We synthesized biotinylated RNAs comprised of the first 42 nucleotides (nt) of exon P3A using the RiboMAX System (Promega) using a PCR-amplified fragment. The forward primer was 5'-TAATACGACTCACTATAGGG*GGTGACATGGTAGATCTGCCA*-3', where T7 promoter is underlined and nucleotides annealed to the reverse primer are italicized. The reverse primers were: 5'-CAAAGTCACGCAGCTGGGGCG*TGGCAGATCTACCATGTCACC*-3' for wild-type, and 5'-CAAAGTCACGCAGCTGGGGTG*TGGCAGATCTACCATGTCACC*-3' for mutant (αP3A23'G>A), where nucleotides annealing to the forward primer are italicized. We synthesized a scrambled (scr) probe as a control, in which nucleotide sequences were scrambled while keeping the nucleotide composition the same. The reverse primer for the scrambled RNA was: 5'-GGCTCGACGAGAGCACGGTTCA*GGCAGATCTACCATGTCACC*-3'. Similarly, we synthesized artificially deleted mutant probes of Δ20-21, Δ24-26, and Δ20-26 by introducing 2–7 nt deletions in the reverse primers.

Mass spectrometry

For tandem mass analysis, we used an LCQ Advantage Mass Spectrometry System (Thermo Finnigan). Samples were injected onto the Paradigm MS4 HPLC System equipped with a Magic C18AQ column of 0.1 mm in diameter and 50 mm in length (Michrom BioResources). Reversed-phase chromatography was performed with a linear gradient (0 min, 5% B; 45 min, 100% B) of solvent A (2% acetonitrile with 0.1% formic acid) and solvent B (90% acetonitrile with 0.1% formic acid) at an estimated flow rate of 1 μl/min. Ionization was performed with an ADVANCE Captive Spray Source (Michrom BioResources) with a capillary voltage at 1.7 kV and temperature of 150 ˚C. A precursor ion scan was carried out using a 400–2000 mass to charge ratio (m/z) prior to MS/MS analysis.

siRNA knockdown and minigene splicing

Cells were plated 24 h before transfection in six-well culture plates (1.5×10^5 cells/well). The transfection reagent included each siRNA duplex at a final concentration in culture medium of 30 nM, 1μl of Lipofectamine 2000 (Invitrogen), and 500 ng of the pSPL3 minigene in 100 μl DMEM. Three days after incubation at 37 ˚C, the cells were harvested and were subjected to

immunoblotting analysis to check protein expression. Total RNA was also isolated from the harvested cells and RT-PCR was performed for splicing analysis.

Tethered function assay of hnRNP L and hnRNP LL

The human *HNRNPL* cDNA clone, encoding 18 to 589 codons, was purchased (clone ID 6174088, Open Biosystems). We introduced the Kozak consensus sequence (5'-CACCATGGAG-3') before codon 18 by PCR, where the initial ATG and second GAG codons are underlined. The amplicon was subcloned into a CMV-based expression vector, pcDNA3.1/V5-His TOPO (Invitrogen) to obtain pcDNA-hnRNP L plasmid. For hnRNP LL, we purchased the human *HNRPLL* cDNA clone (clone ID 3502860, Open Biosystems) that encodes the full-length hnRNP LL covering codons 1 to 542. We similarly cloned cDNA in pcDNA3.1/V5-His TOPO vector to obtain pcDNA-hnRNP LL plasmid.

To construct MS2-tagged fusion protein of hnRNP L, we excised the MS2-coat protein cDNA from the MS2-tagged hnRNP H construct[9], and subcloned it into XhoI and XbaI sites of pcDNA-hnRNP L to obtain pcDNA-MS2-hnRNP L plasmid. We similarly introduced the MS2-coat protein cDNA into NotI and XbaI sites to produce pcDNA-MS2-hnRNP LL. For both constructs, MS2-coat protein cDNA was fused to the C-terminal end of hnRNPs L and LL. We also introduced only the MS2-coat protein cDNA in pcDNA3.1/V5-His TOPO vector to obtain a control plasmid (pcDNA-MS2). We introduced the nuclear localization signal (NLS) of the SV40 large T-antigen just downstream of the start codon of each effector construct by the QuikChange site-directed mutagenesis kit as previously described[9].

We made deletion mutants of hnRNP L from pcDNA-MS2-hnRNP L plasmid. We first amplified two PCR fragments from the full-length pcDNA-MS2-hnRNP L: one upstream of the deletion and the other downstream of the deletion. We introduced EarI restriction sites spanning the deletion. EarI is a type III restriction enzyme and cleaves outside of the recognition sequence, so that the artificial recognition sequence of CTCTTC is removed from the final construct. We ligated the two PCR products and subcloned into pcDNA3.1/V5-His TOPO vector.

We also constructed an insertion mutant of hnRNP LL using the megaprimer method[10] from pcDNA-MS2-hnRNP LL plasmid. We inserted the proline-rich region (PRR) of hnRNP L (codons 335–381) into hnRNP LL after codon 333. At first, we PCR-amplified the PRR of hnRNP L with the primers carrying complementary sequences to hnRNP LL where the PRR is being inserted. The PCR amplicon was used as a megaprimer for the QuikChange site-directed mutagenesis kit to introduce the PRR into pcDNA-MS2-hnRNP LL to obtain

pcDNA-MS2-hnRNP LL-PRR plasmid. We introduced the nuclear localization signal (NLS) of the SV40 large T-antigen immediately after the start codon of each mutant construct as described above. We sequenced the entire inserts of all the clones to ensure absence of PCR artifacts.

***In vitro* splicing and spliceosomal E complex assays**

Templates for E3P3A (wt and mut) and P3AE4 (wt and mut) pre-mRNAs were PCR-amplified from pRBG4 minigenes (wt and mut) and subcloned into pGEM-T Easy vector (Promega). For P3AE4 (wt and mut), PCR products were amplified using primer pairs 5'-CACCATGGGTGACATGGTAGATCTGCCAC-3' and 5'-GCTGCAATAAACAAGTTGACGGTATC-3'. For E3P3A (wt and mut), the length of intron was shortened while keeping 137 nt downstream of exon 3 and 137 nt upstream of exon P3A. We first amplified two PCR fragments from pRBG4 minigenes (wt and mut) using primer pairs: 5'-CACCATGGATGAAGTAAATCAGATCGTGAC-3' and 5'-CATGCTTTCACCATC-3' for the 5' segment; and 5'-GATGGTGAAAGCATGGATTACAGGCATGAGC-3' and 5'-CTCATTCTGCAGATG-3' for the 3' segment, where complementary sequences spanning the deletion are underlined. The two PCR products were then used as primers for overlap-extension PCR to produce E3P3A (wt and mut) and subcloned into pGEM-T Easy vector.

Templates for iP3Ai (wt and mut) pre-mRNAs were amplified from pRBG4 minigenes using primer pairs 5'-GATGTTGCCTGCTTGAG-3' and 5'-AGGGTGATTACTGACCTCATTC-3', and subcloned into pGEM-T Easy vector.

For a plasmid containing MS2 hairpins, a DNA fragment containing three MS2 hairpins was generated by overlap-extension PCR using the following primers, 5'-GGGAAGCTTGGATCCGTACACCATCAGGGTACGAGAGCTC*CCCGGGCGTACACCATCAGGGTACGACTGCAG*-3', 5'-GGGGAATTCTCTAGACGTACCCTGATGGTGTACGGTCGAC*CTGCAGTCGTACCCTGATGGTGTACGCCCGGG*-3'; where HindIII, BamHI, and EcoRI sites are indicated by single, double, and dotted underlines, respectively, and complementary nucleotides are shown in italics. This PCR product was subcloned into the HindIII-EcoRI sites of pSP64 vector (Promega) to generate pSP64-MS2 plasmid. DNA fragments of iP3Ai-wt and iP3Ai-mut were amplified by PCR from pRBG4 minigenes using the following primers: 5'-ATTTAGGTGACACTATAGAAGCTTGTTCACACTAGGTCTTTCTCC-3', 5'-CCCTGATGGTGTACGGATCCAGGGTGATTACTGACCTCATTCT-3'; where HindIII

and BamHI sites are indicated by single and double underlines, respectively, and cloned into pSP64-MS2 to make pSP64-iP3Ai-wt-MS2 and pSP64-iP3Ai-mut-MS2 plasmids.

The MS2 hairpins (BamHI-EcoRI DNA fragment of pSP64-MS2) were introduced into the BamHI-EcoRI sites of pSP64-HβΔ6 encoding human β-globin exon 1-intron 1-exon 2[11] to make pSP64-HβΔ6-MS2 plasmid. pGEM-T Easy vectors carrying E3P3A-wt/mut, P3AE4-wt/mut, iP3Ai-wt/mut were linearized by NotI, NotI, and SalI, respectively. pSP64 vectors carrying iP3Ai-wt/mut-MS2 and HβΔ6-MS2 were linearized by EcoRI. The linearized vectors were used as templates for *in vitro* transcription using RiboMAX Large Scale RNA Production System (Promega).

Supplementary References

1 Bottema, C. D. & Sommer, S. S. PCR amplification of specific alleles: rapid detection of known mutations and polymorphisms. *Mutat. Res.* **288**, 93-102 (1993).

2 Luther, M. A. *et al.* A Muscle Acetylcholine-Receptor Is Expressed in the Human Cerebellar Medulloblastoma Cell-Line Te671. *J. Neurosci.* **9**, 1082-1096 (1989).

3 Ohno, K. *et al.* Congenital myasthenic syndrome caused by decreased agonist binding affinity due to a mutation in the acetylcholine receptor epsilon subunit. *Neuron* **17**, 157-170 (1996).

4 Schoepfer, R., Luther, M. & Lindstrom, J. The human medulloblastoma cell line TE671 expresses a muscle-like acetylcholine receptor. Cloning of the alpha-subunit cDNA. *FEBS Lett.* **226**, 235-240 (1988).

5 LEE, B. S., Gunn, R. B. & Kopito, R. R. Functional Differences among Nonerythroid Anion-Exchangers Expressed in a Transfected Human Cell-Line. *J. Biol. Chem.* **266**, 11448-11454 (1991).

6 Ohno, K., Milone, M., Shen, X. M. & Engel, A. G. A frameshifting mutation in CHRNE unmasks skipping of the preceding exon. *Hum. Mol. Genet.* **12**, 3055-3066 (2003).

7 Kozak, M. An analysis of 5'-noncoding sequences from 699 vertebrate messenger RNAs. *Nucleic Acids Res* **15**, 8125-8148 (1987).

8 Sahashi, K. *et al.* In vitro and in silico analysis reveals an efficient algorithm to predict the splicing consequences of mutations at the 5' splice sites. *Nucleic Acids Res.* **35**, 5995-6003 (2007).

9 Masuda, A. *et al.* hnRNP H enhances skipping of a nonfunctional exon P3A in CHRNA1 and a mutation disrupting its binding causes congenital myasthenic syndrome. *Hum. Mol. Genet.* **17**, 4022-4035 (2008).

10 Ohno, K. *et al.* Myasthenic syndromes in Turkish kinships due to mutations in the acetylcholine receptor. *Ann. Neurol.* **44**, 234-241 (1998).

11 Krainer, A. R., Maniatis, T., Ruskin, B. & Green, M. R. Normal and mutant human beta-globin pre-mRNAs are faithfully and efficiently spliced in vitro. *Cell* **36**, 993-1005 (1984).

Supplementary Figure Legends

Supplementary Figure S1. Conservation of glycine at codon 421 in the M4 transmembrane domain of AChR and the family pedigree of the patient with CMS. (a) Alignment of the M4 transmembrane domain of human AChR α, β, δ, γ, and ε subunits. (b) Alignment of the M4 transmembrane domain of AChR α subunits of other animal species. Conserved glycines are boxed in (a) and (b). (c) Family analysis shows that each mutation is heteroallelic and recessive in the patient. The patient is indicated by an arrow and a shaded symbol. Half-shaded squares (males) and circles (female) represent asymptomatic heterozygous carriers.

Supplementary Figure S2. Additional information to Figure 3. (a) Schematic of *CHRNA1* gene and probes at exonic positions 1–42 used for RNA affinity purification. (b) Immunoblots (IB) probed with candidate splicing *trans*-factors. NE indicates 10% of nuclear extract used. (c) Immunoblotting of mock-, hnRNP L-, and hnRNP LL-depleted SH-SY5Y nuclear extracts showing efficient removal of hnRNPs L and LL.

Supplementary Figure S3. Lack of off-target effects of siRNAs and lack of off-target effects of MS2 coat protein-fused constructs. (a) RT-PCR of wild-type (wt) and mutant (mut) pSPL3 minigenes in SH-SY5Y cells treated with siRNA against control (siCt), hnRNP L (siL), and hnRNP LL (siLL) under expression of siRNA-resistant cDNA for hnRNPs L (si-res L) and LL (si-res LL). Immunoblotting shows expression of His-tagged si-res L and LL proteins. Exon P3A inclusion driven by siL on wild-type pre-mRNA is efficiently counteracted by si-res L, and exon P3A skipping driven by siLL on mutated pre-mRNA is counteracted by si-res LL (see Fig. 3e). (b) RT-PCR of pSPL3 minigenes without MS2 sequence (pSPL3-nonMS2) in SH-SHY5Y cells co-transfected with the indicated effectors. HnRNPs L and LL are fused to the MS2 coat protein (MS2-L and MS-LL, respectively). Immunoblotting shows expression of these His-tagged effectors in the nuclear lysate of SH-SY5Y cells. Bars and lines represent mean and SD, respectively, of three independent experiments for all the panels.

Supplementary Figure S4. HnRNP L and PTB cooperatively suppress inclusion of exon P3A. RT-PCR of wild-type pSPL3 minigene in SH-SY5Y cells treated with the indicated siRNA. Immunoblotting (IB) shows effective repression of hnRNP L and PTB. siCt, siL, and siPTB

indicate control-, hnRNP L-, and PTB-targeted siRNA, respectively. Bars and lines represent mean and SD, respectively, of three independent experiments.

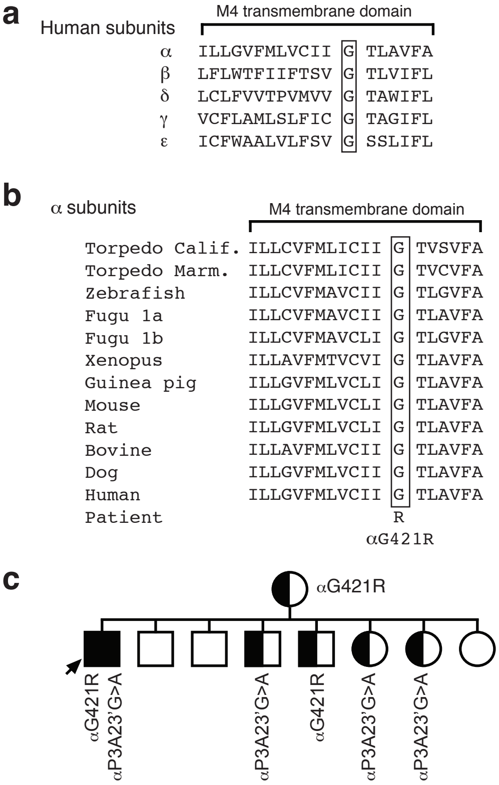

Supplementary Figure S1

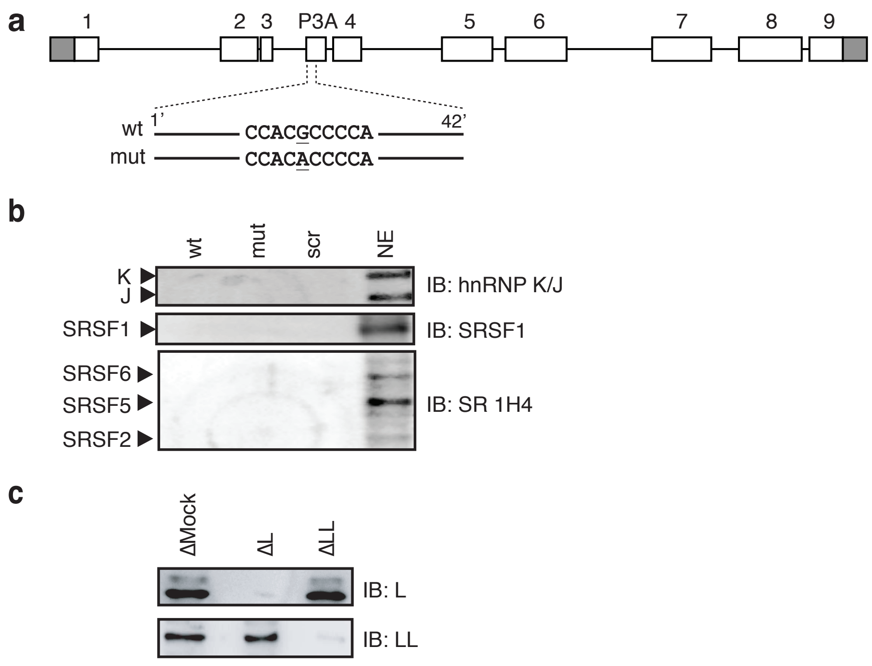

Supplementary Figure S2

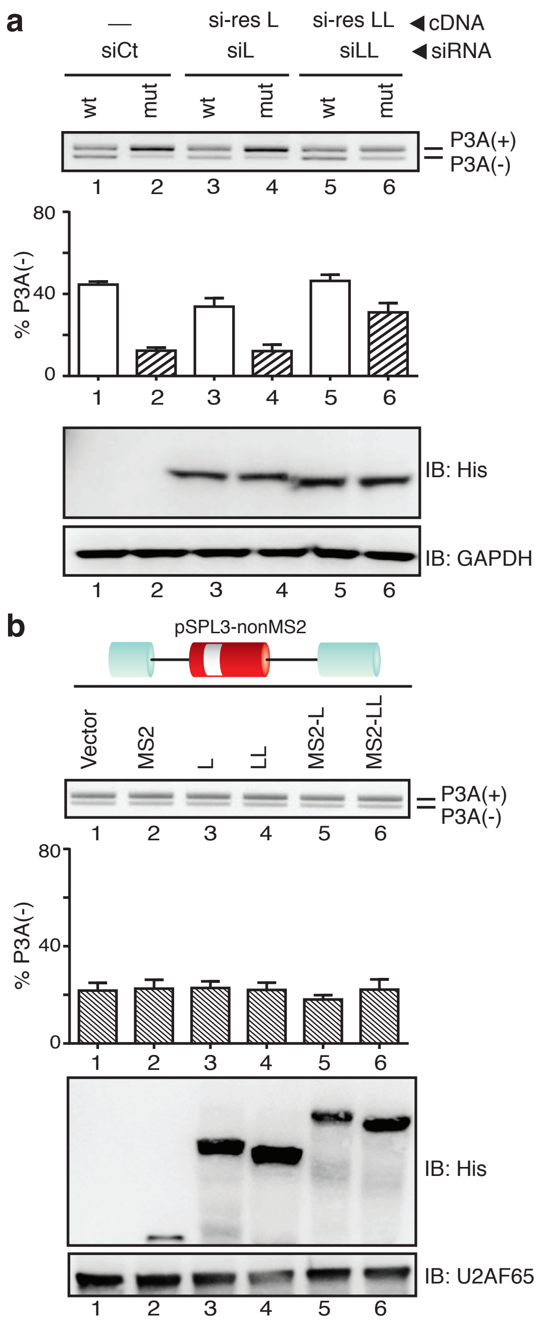

Supplementary Figure S3

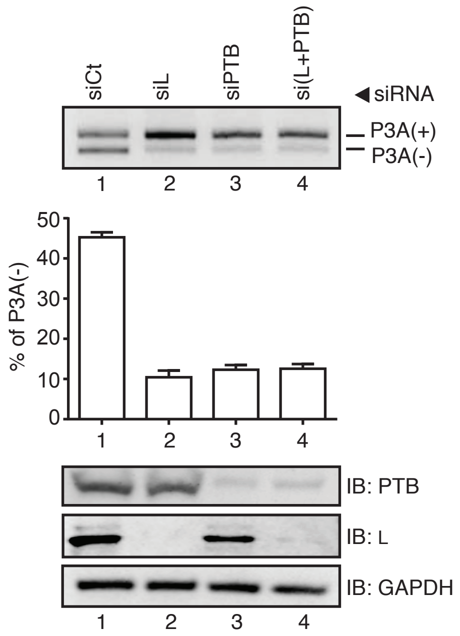

Supplementary Figure S4
